# Linking periodontal pathogens with endoperio lesions

**DOI:** 10.6026/973206300200583

**Published:** 2024-05-31

**Authors:** Sakshi Malik, K. Sai Priyanka, Rinkee Mohanty, Anas Abdul Khader, Shekhar Prasad, Souradeep Dey, Jeethu John Jerry

**Affiliations:** 1 Department of Pediatric and Preventive Dentistry, Daswani Dental College and Research Centre, Kota, Rajasthan, India; 2 Periodontist, Hyderabad, Telangana, India; 3 Department of Periodontics, Institute of Dental Sciences, SOA Deemed to be university, Bhubaneswar, Odisha, India; 4 Department of Periodontology and Implant Dentistry, College of Dentistry, Qassim University, Kingdom of Saudi Arabia; 5 Department of Prosthodontics and Implantology, Maharaja Ganga Singh Dental College and Research Centre, Sri Ganganagar, Rajasthan, India; 6 Intern, Kalinga Insititute of Dental Sciences, KIIT University, Bhubaneswar, Odisha, India; 7 Department of Periodontology, Malabar Dental College and Research Centre, Edappal, Malapuram, Kerala, India

**Keywords:** Dentinal tubules, Endodo-perio lesion, *Porphyromonas gingivalis*, *Tannerella forsythia*

## Abstract

Endodontic-periodontal diseases pose difficulties for the practitioner in diagnosing and predicting the success of the affected teeth. Therefore, it is of interest to correlate between periodontal infections and endodontic periodontal disorders. 50 patients of both sexes were included in this study. 28 of the 50 patients were men and 22 were women. Participants with a history of endodontic and periodontal lesions on the same tooth were chosen. A polymerase chain reaction experiment was carried out and relationships were formed. Data shows that isolates of *Porphyromonas gingivalis*, *Aggregatibacter actinomycetemcomitans* and *Tannerella forsythia* were identified in 91% of the periodontium, 12% of the endodontium, and 51% of the endodontium, respectively. Targeted bacterial species were associated with periodontal and endodontic disorders that occurred concurrently. Therefore, it is plausible to speculate that dentinal tubules serve as a channel for the dissemination of microorganisms.

## Background:

It is difficult for the practitioners to diagnose and predict the success of the affected teeth with endodontic-periodontal diseases. Periodontal tissues and dental pulp have has close association. The development and evolution of such lesions are significantly influenced by a number of contributing variables, including trauma, perforations, root resorptions, and dental abnormalities, as well as etiologic factors including fungi, bacteria, and viruses [1,2]. In regions without cementum, exposed dentinal tubules may function as a connecting route between the periodontal ligament and the pulp [1]. There may also be a direct line of contact between the pulp and the periodontium through the accessory canals in the furcation area of molar. The main channel of contact between the pulp and the periodontium is the apical foramen [1, 3]. It has been found that bacteria can enter through dentinal tubules, and the endodontic-periodontal pathway niche is susceptible to endodontic infection [4]. Permeability to the dentinal tubules can be influenced by various factors, including the size, adhesiveness, and motility of the bacteria [5]. The dentinal tubule exposed to the periodontal ligament is covered by cement [6]. Because the periodontium and endodontium are closely connected tissues, illnesses affecting one may affect the other. It can occasionally be challenging to differentiate between endodontic and periodontal illnesses, but getting the diagnosis right is essential to getting the right treatment [2, 7]. The cornerstone of treatment for periodontal diseases is scaling and root planing, which involves removing contaminated cementum [4]. The aetiology and accurate diagnosis of each unique illness determine the treatment and prognosis of endodontic-periodontal diseases [1]. Therefore, it is of interest to explore the correlation between periodontal infections and endodontic periodontal disorders.

## Materials and Method:

The Department of Periodontology and Endodontics carried out this investigation. The institutional ethical committee granted ethical approval. Written informed consent was obtained from each patient. Fifty patients, aged 25-55, with a single rooted tooth exhibiting endodontic or periodontal disease, and patients with periodontal pockets less than 6 mm, were included in this research. Participants who had a history of antibiotic usage within the last three months, had an aggressive case of periodontitis, had radiographic evidence of endodontic-periodontal contact, or had a fistula were excluded. Patient information was entered into case history performa, including name, age, gender, and other details. To diagnose endodontic-periodontal lesions, intraoral periapical radiographs were performed on each patient. Two to three paper points were placed into periodontal pockets and left there for one minute each in order to obtain periodontal samples. Two to three sterile paper points were placed into the root canal and left there for one minute in order to collect samples from a tooth that had a single root canal. After that, the paper points were put into cryotubes with one millilitre of TE buffer inside of them. Samples were frozen right away and stored at -80°C.

## Extraction of DNA:

Prior to the extraction of DNA, frozen endodontic and periodontal specimens were suspended and allowed to settle at room temperature. The Qiamp DNA small kit (Qiagen, Hilden, Germany) was used for this experiment. The UV micro spectrophotometer 1240 was used to measure the concentration and purity of the DNA. Through the use of an indirect evaluation method, the precise number of DNA copies within the bacterium was determined. The American Type Culture Collection (ATCC; Manassas, Virginia) provided *Aggregatibacter actinomycetemcomitans* ATCC 29523, *Tannerella forsythia* ATCC 43037, and *Porphyromonas gingivalis* ATCC 33277. Using the student's t test at a significance level of 0.05, the collected data was statistically assessed using the SPSS software programme, version 23.0.0 (SPSS, Chicago, Illinois).

## Results:

Out of 50 patients in the current study, 22 were women and 28 were men. Table 1 lists the species found in the periodontium and endodontium. In 93% of the endodontium, 91% of the periodontium, 71%, and 48% of the *P. gingivalis* isolated from the endodontium and periodontium were found to harbour *Tannerella forsythia*. A. In 51% of the periodontium and 12% of the endodontium, Actinomycetemcomitans was identified. The total number of microorganisms found in the endodontium and periodontium for a particular tooth did not change significantly (Table 1).

The presence of *T. forsythia* and *P. gingivalis* (r = 0.489 and P = 0.02), *P. gingivalis* and *A. actinomycetemcomitans* (r = 0.607 and P = 0.01), and *T. forsythia* and *A. actinomycetemcomitans* (r = 0.476 and P = 0.03) are all significantly correlated, according (Table 2).

## Discussion:

In 1964, Simring and Goldberg made the initial connection between periodontal and pulpal disease. Lesions resulting from inflammatory products present in the pulpal tissues and periodontium in some manner have been referred to as "*perio-endo*" lesions [4]. The prognosis for endo-perio lesions varies, and they are difficult to identify and treat [8]. Endodontic-periodontal disorders are influenced by the complex microbiota of periodontal pockets (PPs) and the microorganisms present in root canals (RCs) [9]. Both periodontal and endodontic therapy is necessary for the majority of periodontal diseases with subsequent endodontic involvement [4]. The current investigation revealed that the number of targeted pathogens in the endodontium and periodontium of the same tooth did not differ statistically. The optimal natural conditions for bacterial development in periodontal pockets are provided by the significant quantifiable correlations of *T. forsythia*, F. nucleatum, and *P. gingivalis* quantification in these pockets, according to the results of the Pearson test. After comparing *A. actinomycetemcomitans* and *T. forsythia*, *P. gingivalis* and *A. actinomycetemcomitans*, and *A. actinomycetemcomitans* and *T. forsythia*, we found a fundamental connection between the bacteria in endodontic-peridontal disease.

Periodontal disease may be brought on by bacteria that enter the root canal through the apical foramen, according to Abbot and Salgado [10]. By spreading through these channels or dentinal tubules, these bacteria may aid in the development of periodontitis [6]. The frequency of Endo-Perio lesions in individuals belonging to a known demographic was assessed by Altaf *et al.* They came to the conclusion that Endo-Perio lesions affect a sizable fraction of the patient population [2]. Potential prognostic variables for endodontic-periodontal lesions were identified by Wong *et al.* According to their findings, the likelihood of success in treating endodontic-periodontal lesions may be influenced by a history of periodontal disease [11]. The connection between periodontal infections and endoperio lesions was evaluated by Kalvani *et al.* According to their statement, dentinal tubules serve as a channel for the transmission of germs [6]. A research by Terlemez *et al.* revealed that periodontitis reduces pulp volume by about 20% [12]. Endo-perio lesions are rather common in multirooted teeth, such as molars and premolars, because these teeth have several accessory canals. It's possible that the existence of coronal and cervical dentinal tubules provides a means of dual site infection maintenance and dissemination. Both endo perio therapies carried out right away result in shorter treatment times and higher patient compliance [13]. Regardless of whether the disorders manifest as separate or combined lesions, full healing from both conditions is necessary for the success of periodontal and endodontic therapies [12]. It has been noted that successful endodontic therapy combined with definitive periodontal therapy is the only way to address persistent periodontal disease [14]. In patients having endodontic treatment with either mineral-trioxide or gutta-percha, the use of diode laser, the management of PRF and titanium-prepared PRF, and the deployment of bone grafts appear to be an adequate strategy [15]. Iatrogenic endodontic-periodontal lesions can be identified and treated more effectively with the use of a systematic diagnosis process [16]. The drawback of the present study is smaller sample size and use of only single rooted tooth. Further researches are needed to validate the findings.

## Conclusion:

Data shows that targeted bacterial species are associated with periodontal and endodontic disorders that occurred concurrently. Thus, dentinal tubules could serve as a conduit for bacterial dissemination. Hence, iatrogenic endodontic-periodontal lesions can be identified and treated more effectively with the use of a systematic diagnosis process.

## Figures and Tables

**Table 1:** Detected species in endodontion and periodontium

**Detected species**	**Endodontium (%)**	**Periodontium (%)**	**P**
*Tannerella forsythia*	93	91	
*Porphyromonas gingivalis*	71	48	0.021
*Aggregatibacter actinomycetemcomitans*	12	51	

**Table 2:** Pearson's correlation between bacterial species of same tooth

**Detected species**	**r**	**P**
*Tannerella forsythia and Porphyromonas gingivalis*	0.489	0.02
*Porphyromonas gingivalis and Aggregatibacter actinomycetemcomitans*	0.607	0.001
*Tannerella forsythia and Aggregatibacter actinomycetemcomitans*	0.476	0.03

## References

[1] Rotstein I (2006). Endodontic Topics..

[2] Altaf A (2019). International Journal of Applied Dental Sciences..

[3] Anderson AC (2013). PLoS One..

[4] Das AC (2020). J Family Med Prim Care..

[5] Wang HL (2002). Pathways of the Pulp. 8th ed..

[6] Kalvani Het (2022). Int J Oral Care Res..

[7] Tayal A (2021). IP Indian Journal of Conservative and Endodontics.

[8] Makeeva MK (2020). Clin Cosmet Investig Dent..

[9] Lopes EM (2021). Microorganisms..

[10] Abbot PV, Salgado JC (2009). Aust Dent J..

[11] Wong et (2024). Clin Exp Dent Res..

[12] Terlemez A (2018). J Endod..

[13] Tewari S (2018). J Oral Biol Craniofac Res..

[14] Saha AP (2018). Int J Appl Dent Sci..

[15] Ardila CM, Vivares-Builes AM (2022). Int. J. Environ. Res. Public Health..

[16] Alfawaz Y (2017). World Journal of Dentistry..

